# Spectral properties of bacteriophytochrome *AM1_5894* in the chlorophyll *d*-containing cyanobacterium *Acaryochloris marina*

**DOI:** 10.1038/srep27547

**Published:** 2016-06-10

**Authors:** Patrick C. Loughlin, Zane Duxbury, Tendo T. Mukasa Mugerwa, Penelope M. C. Smith, Robert D. Willows, Min Chen

**Affiliations:** 1 School of Biological Sciences, University of Sydney, NSW 2006, Australia; 2 Department of Chemistry and Biomolecular Sciences, Macquarie University, NSW 2109, Australia

## Abstract

*Acaryochloris marina*, a unicellular oxygenic photosynthetic cyanobacterium, has uniquely adapted to far-red light-enriched environments using red-shifted chlorophyll *d*. To understand red-light use in *Acaryochloris*, the genome of this cyanobacterium was searched for red/far-red light photoreceptors from the phytochrome family, resulting in identification of a putative bacteriophytochrome *AM1_5894*. AM1_5894 contains three standard domains of photosensory components as well as a putative C-terminal signal transduction component consisting of a histidine kinase and receiver domain. The photosensory domains of AM1_5894 autocatalytically assemble with biliverdin in a covalent fashion. This assembled AM1_5894 shows the typical photoreversible conversion of bacterial phytochromes with a ground-state red-light absorbing (Pr) form with λ_BV max_[Pr] 705 nm, and a red-light inducible far-red light absorbing (Pfr) form with λ_BV max_[Pfr] 758 nm. Surprisingly, AM1_5894 also autocatalytically assembles with phycocyanobilin, involving photoreversible conversion of λ_PCB max_[Pr] 682 nm and λ_PCB max_[Pfr] 734 nm, respectively. Our results suggest phycocyanobilin is also covalently bound to AM1_5894, while mutation of a cysteine residue (Cys11Ser) abolishes this covalent binding. The physiological function of AM1_5894 in cyanobacteria containing red-shifted chlorophylls is discussed.

Photoreceptors detect changes in light quality, and in response, they trigger cellular signals that regulate downstream physiological processes. Phytochromes are a group of photoreceptors that are particularly important to photosynthetic organisms because they detect photosynthetically active radiation, having absorption maxima similar to chlorophylls^1^. Phytochromes, which were first characterized in plants, act as red/far-red photoreceptors in two interconvertible forms: a red light-absorbing form (Pr) and a far-red light-absorbing form (Pfr). The ground state Pr form converts to the activated state Pfr upon illumination with red light. Extended dark or illumination with far-red light converts the Pfr form back to the Pr form. Phytochromes have since been identified in fungi, photosynthetic and non-photosynthetic bacteria^2^.

Phytochromes are modular proteins that typically consist of an N-terminal photosensory core composed of PAS (a domain named after the proteins: period circadian protein, aryl hydrocarbon receptor nuclear translocator protein, and single-minded protein); GAF (a domain, named after cGMP-specific phosphodiesterases, adenylyl cyclases, and FhlA proteins) and PHY (phytochrome) domains, as well as a C-terminal signal transduction domain that is commonly a histidine kinase^3^. The phytochrome superfamily has been subdivided into a number of clades: plant phytochromes (Phys), cyanobacterial phytochromes (Cphs), bacteriophytochromes (BphPs), fungal phytochromes (Fphs) and the more distantly related Phy-like phytochromes (also known as cyanobacteriochromes or CBCRs)^2^,^4^. The binding of different linear tetrapyrroles (or bilins), as well as their manner of binding, allows phytochromes from different organisms to detect changes in light conditions relevant to a particular organism. All bilins are derived from a heme molecule, converted to the linear tetrapyrrole biliverdin IXα (BV) by heme oxygenase. This BV can be modified to 3-E-phytochromobilin (PΦB) or 3-Z-phycocyanobilin (PCB) by bilin reductases. While BphPs typically bind the simplest linear tetrapyrrole (BV) via a conserved cysteine (Cys) residue in the PAS domain *in vivo*^5^, Phys and Cphs bind PΦB and PCB, respectively, via a Cys residue in the GAF domain^6^. In addition to Cphs, some cyanobacteria possess cyanobacterial bacteriophytochromes (cBphPs). These cBphPs are phylogenetically similar to Cphs, but like BphPs they have a conserved Cys residue in their PAS domain, and lack a chromophore-binding Cys residue in their GAF domain^2^. There is evidence to support that a highly conserved His residue in the GAF domain is important for chromophore attachment in some BphPs^7^. However, crystallographic data suggest that this highly conserved His residue is involved in a network of hydrogen bonding in the chromophore-binding pocket^8^, rather than acting as a covalent ligation site. Atypically, some BphPs have their ground state absorption maxima at a longer wavelength than their light-activated state (*i.e*., the ground state is the Pfr form), and are called bathyphytochromes (Bathy BphPs) accordingly^5^,^9^,^10^.

CBCRs are the most structurally diverse subgroup of the phytochromes, often missing highly conserved amino acid residues found in other phytochromes; they can respond to nearly the entire visible light spectrum^11^. CBCRs often contain multiple GAF domains with Cys residues, which can either reversibly or irreversibly bind PCB^1^,^4^,^12^,^13^. The evolution of such a diverse family of photoreceptors in cyanobacteria as well as the wide spectral range detected by these photoreceptors highlights the importance of light quality for cyanobacteria. Clearly, this reflects the fact that cyanobacteria are photoautotrophs that harvest light for photosynthesis at a number of different wavelengths, the amounts of which are dependent on the level they inhabit in a water column and competition from other photosynthetic organisms^14^. Recent research has demonstrated that phytochromes from eukaryotic algae that inhabit similar ecological niches to cyanobacteria also have evolved complicated photocycles, beyond typical red/far-red light switching mechanisms^15^.

*Acaryochloris marina* (*Acaryochloris*) is a cyanobacterium that uses chlorophyll *d* (Chl *d*) instead of Chl *a* in the reaction centre of both photosystems^16^,^17^. Recently, two additional cyanobacterial species have been shown to contain trace amounts of Chl *d*^18^,^19^. However, the function of Chl *d* in these organisms has not been established. The type species, *Acaryochloris* MBIC11017, was discovered as a symbiont of a colonial ascidian in the Pacific Ocean^20^. Subsequently, several ecotypes of *Acaryochloris* have been discovered in niche environments around the world that are enriched in red/far-red light^21^,^22^,^23^,^24^,^25^,^26^,^27^,^28^. Chl *d* has a red-shifted absorbance^29^, which allows *Acaryochloris* to exploit such niche environments^22^. The Q_Y_ peaks of Chl *a* and Chl *d in vivo* are approximately 675 nm and 712 nm, respectively^30^, giving Chl *d* an absorption maximum intermediate to Chl *a* and bacteriochlorophylls^31^. *Acaryochloris* captures light energy in its reaction centres using two discrete light-harvesting systems: (1) a Chl *d*-binding antenna predominantly of Chl *d* with small amounts of Chl *a*^32^; and (2) a phycobiliprotein antenna that is a simpler version of phycobilisomes found in other cyanobacteria^33^,^34^. *Acaryochloris* is able to regulate these antennas in response to changing light quality or intensity in a manner similar to complementary chromatic adaptation^30^,^35^,^36^. The newly identified far-red/green reversible cyanobacteriochrome AM1_1870 indicates that *Acaryochloris* has a complementary light regulatory system to different environmental conditions^37^. In addition, a number of characterised CBCRs with photocycles ranging from blue to far-red absorption maxima (up to absorption peak at 706 nm) suggest *Acaryochloris* may use these to signal responses to different light conditions^38^,^39^.

In this work, a putative phytochrome gene, *AM1_5894*, was identified in the *Acaryochloris* genome using BLAST and sequence analysis^40^. Sequence comparison revealed that AM1_5894 is part of a small cluster of cyanobacterial BphPs, which are related to BphPs from non-photosynthetic and anoxygenic photosynthetic bacteria, but distinct from the typical Cphs in Chl *a*-containing cyanobacteria. We demonstrate that heterologously expressed *AM1_5894* covalently binds the chromophores PCB and BV, and undergoes photoconversion between a Pr and Pfr form *in vitro*. The reconstituted BV-AM1_5894 has a spectral profile in its Pr/Pfr state (λ_BV max_[Pr] 705 nm and λ_BV max_[Pfr] 758 nm). Reconstitution of AM1_5894 with PCB blue shifts the absorption maxima by approximately 23 nm (λ_PCB max_[Pr] 682 nm and λ_PCB max_[Pfr] 734 nm), broadening the AM1_5894 light detection range, when it acts as a photoreceptor in *Acaryochloris*. Furthermore, covalent attachment of both chromophores is abolished when the presumed adduct (Cys11) is mutated. We show that it is advantageous for *Acaryochloris* to have a BphP instead of a typical Cph protein in its enriched red/far-red light-enriched environment.

## Results

### Protein domain comparison

**Figure 1:**
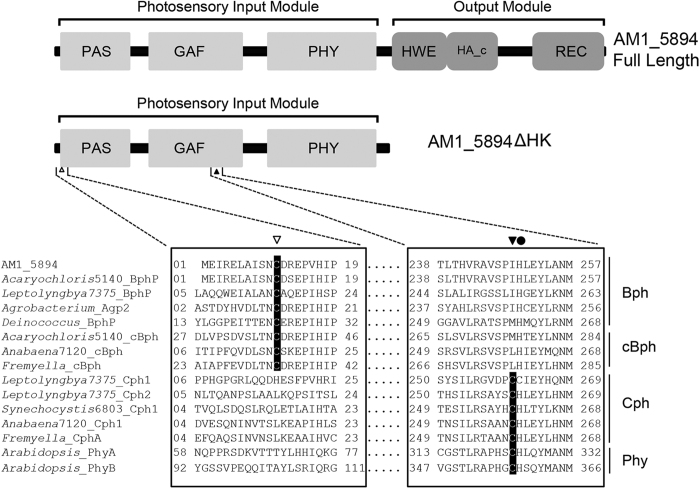
Domain structure of AM1_5894 and alignment of predicted biliverdin (BV) and phycocyanobilin (PCB) binding sites of selected phytochromes. The domain structure of AM1_5894 and the truncated AM1_5894ΔHK used in this study are shown. Alignments highlight the conserved Cys residue in the PAS domain (open triangle; named after period circadian protein, aryl hydrocarbon receptor nuclear translocator protein and single-minded protein) that covalently binds BV in bacteriophytochrome (BphP) and cyanobacterial BV-binding bacteriophytochrome (cBphP) and the conserved Cys residue in the GAF domain (closed triangle; named after cGMP-specific phosphodiesterases, adenylyl cyclases and FhlA proteins) that binds PCB or phytochromobilin (PΦB) in proteins in cyanobacterial phytochromes (Cphs) and plant phytochromes (Phys), respectively. The his residue adjacent this position which is conserved in most phytochromes is also marked (closed circle). HWE (histidine-tyrosine-glutamate -type histidine kinase(HK); HA_c (Histidine kinase-like ATPase c-terminal region); REC (cheY-like receiver domain). For other abbreviations refer to the text. Additional sequence information is given in Supplementary Table 1.

The *AM1_5894* gene comprises 2544 nucleotides and encodes an 847-amino acid protein product, having a predicted molecular weight of 94.2 kDa. The overall domain structure of AM1_5894 is typical of other bacteriophytochromes, with a photosensory core module of PAS-GAF-PHY as well as a C-terminal signal transduction domain, consisting of a histidine kinase; and a receiver domain (Fig. 1). The PAS domain contains a Cys residue at its eleventh position that aligns with the conserved BV-binding Cys of typical BphPs. It also has a His residue (H249) in its GAF domain that is conserved (Fig. 1). The adjacent PCB/PΦB-binding Cys found in the GAF domain of cyanobacterial and plant phytochromes is absent in AM1_5894 (Fig. 1). AM1_5894 is more closely related to BphPs from non-photosynthetic and anoxygenic photosynthetic bacteria than cyanobacterial phytochromes (Cph), based on alignment of their GAF domains^40^. Extending this alignment to include both the GAF and PHY domains confirms that AM1_5894 is phylogenetically separate from other Cphs and cBphPs, apart from the BphPs from another *Acaryochloris* ecotype, CCMEE 5410, and the filamentous cyanobacterium *Leptolyngbya* PCC 7375 (Figure S1). Unusually, the signal output domain of this cluster of three BphPs consists of a HWE (histidine-tyrosine-glutamate)-type histidine kinase and a receiver domain, which is a typical domain structure of Bathy BphPs. Bathy BphPs are most commonly found in Rhizobiales^10^,^41^. Although *Acaryochloris* MBIC 11017 does not contain any Cph-like phytochromes, a number of putative CBCRs, including the recently characterized AM1_1186, AM1_1870 and AM1_1557, are present^37^,^38^,^39^.

### Expression of recombinant AM1_5894

Initial attempts to clone and express the full-length AM1_5894 were largely unsuccessful. A clone with a point mutation in its histidine kinase domain causing a Gly639 → Asp substitution was obtained, although only a small amount of soluble protein showing typical bacteriophytochrome spectral characteristics could be expressed in *Escherichia coli* (Figure S2). Instead, a truncated construct was expressed in *E. coli*. This truncated protein (AM1_5894∆HK) is 508-amino acids in length, compared with the full-length protein of 847 amino acids. AM1_5894∆HK contains PAS, GAF and PHY domains that are essential for chromophore attachment and photochromism^42^,^43^,^44^,^45^, but lacks the output module, including the histidine kinase and receiver domain (Fig. 1). AM1_5894∆HK was His-tag purified almost to homogeneity and used to examine the chromospectral properties of the AM1_5894 holoprotein.

### Chromophore-protein Reconstitution

**Figure 2:**
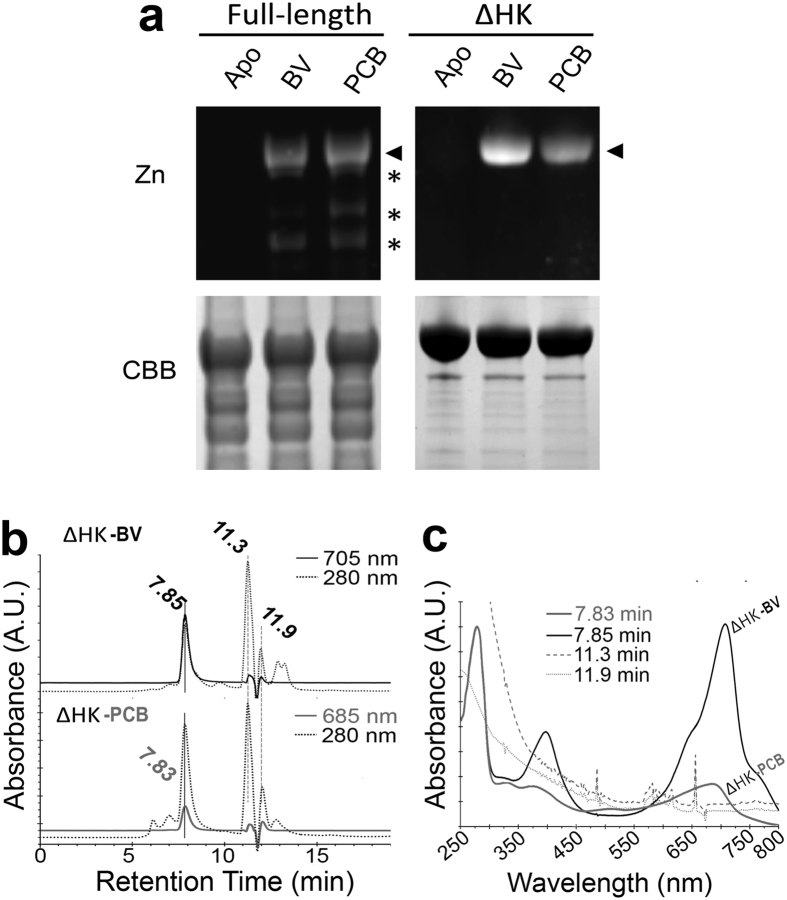
Covalent binding of biliverdin (BV) and phycocyanobilin (PCB) by full length AM1_5894D639G and AM1_5894ΔHK. (**a**) SDS-polyacrylamide gel of either full length AM1_5894D639G or AM1_5894ΔHK after incubation in the absence (Apo) or presence of either BV or PCB. Covalently bound chromophore was detected by Zn induced fluorescence (Zn). Closed arrows mark the fluorescent chromophore-protein complexes. Asterisks mark degradation products of the full length AM1_5894D639G which were not observed for the truncated construct. CBB, Coomassie brilliant blue. (**b**) BioSec2000 purification of assembled AM1_5894ΔHK with BV and with PCB. The assembled chromophore protein complexes have a retention time of approximately 7.8 min (**c**) on-line spectral comparison of BV- and PCB-assembled AM1_5894ΔHK from the BioSec2000 column at indicated retention times.

Based on sequence analysis, AM1_5894 was predicted to covalently bind BV, but not PCB because of a conserved Cys residue in its PAS domain, and lack of a conserved PCB/PΦB-binding Cys residue in its GAF domain (Fig. 1). To confirm this prediction, overexpressed full-length AM1_5894D639G and truncated AM1_5894∆HK were incubated in the absence or presence of either BV or PCB to facilitate autocatalytic attachment of the chromophore. Surprisingly, both reconstituted BV- and PCB-AM1_5894 protein complexes exhibited Zn-induced fluorescence on SDS-polyacrylamide gels (Fig. 2a). This demonstrates that both BV and PCB are covalently bound to AM1_5894 *in vitro*. Due to the low yields and point mutation of our full-length AM1_5894 clone, the truncated construct (AM1_5894∆HK) was used for chromospectral analyses of AM1_5894.

**Figure 3:**
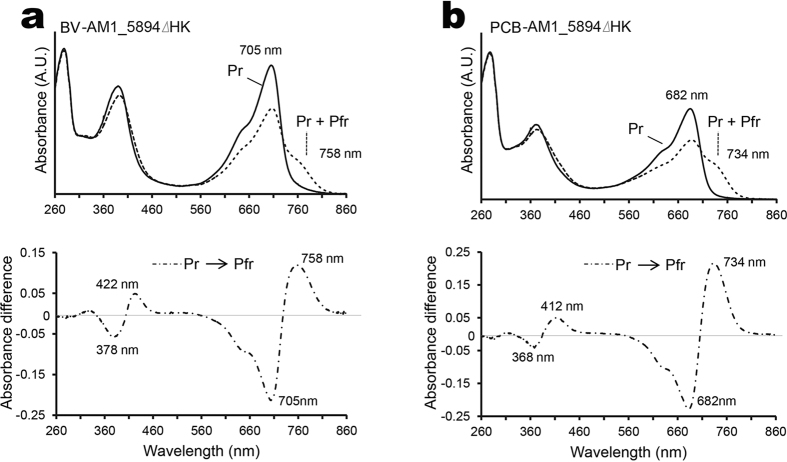
Spectral characterization of biliverdin (BV) and phycocyanobilin (PCB) assembled recombinant AM1_5894ΔHK holoprotein. (**a**) Absorbance spectrum of the BV-AM1_5894ΔHK in its Pr state (solid line) and its Pr + Pfr mixed state (dashed line) following exposure to saturating 777 nm light. The difference spectrum (dash dotted line) is shown below. (**b**) Absorbance spectrum of the PCB-AM1_5894PΔHK in its Pr state (solid line) and its Pr + Pfr mixed state (dashed line) following exposure to saturating 777 nm light. The difference spectrum (dash dotted line) is shown below. Wavelength maxima and minima are indicated.

In order to avoid any bias that may be generated by self-aggregated chromophores, either BV or PCB was added directly to lysed *E. coli* containing overexpressed AM1_5894∆HK before His-tag purification. Further purification was carried out using non-denaturing size exclusion chromatography (Fig. 2b). Online spectra of the chromophore-binding protein complexes confirmed the elution peak at 7.8 min corresponded to the assembled holoproteins, with the same spectra observed *in vitro* (Figs 2c and 3).

### Spectral characterization

**Figure 4:**
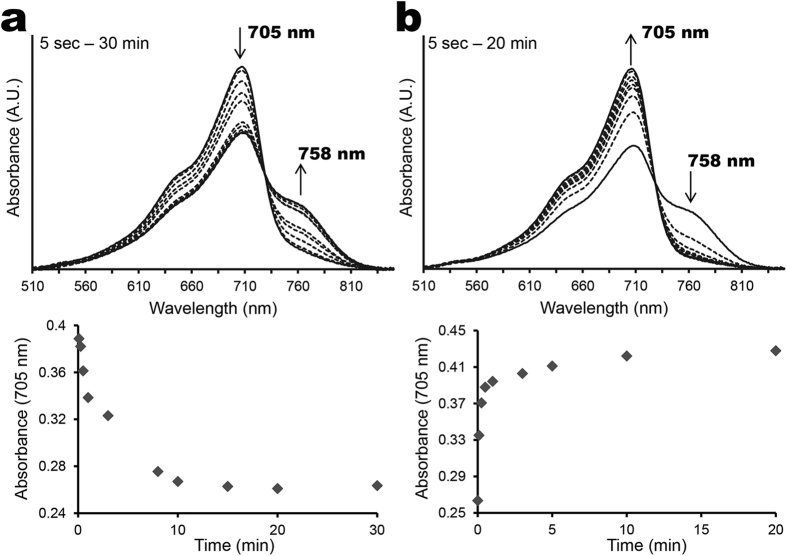
Photoconversion kinetics for BV-AM1_5894ΔHK. (**a**) Pr state BV-AM1_5894ΔHK was illuminated with 665 nm light for the indicated period of time. (**b**) Pfr/Pr state BV-AM1_5894PΔHK was illuminated with 777 nm light for indicated period of time. Δ705 nm is plotted as a measure of photo-switching.

The absorption spectrum of dark-assembled BV-AM1_5894ΔHK resembles that of a typical phytochrome in its Pr state (Fig. 3a). It has two main absorption peaks centred at 380 and 705 nm, with a shoulder at approximately 650 nm (Fig. 3a). Upon illumination with red light (665 nm), there is a reduction in the 705 nm peak, accompanied by the appearance of a longer wavelength peak at 758 nm, which is indicative of the Pfr form (dashed line, Fig. 3a). Illumination of the Pfr form with far-red light (777 nm) or dark incubation causes AM1_5894 to switch back to its Pr state. The difference spectrum of BV-AM1_5894ΔHK Pfr minus Pr demonstrates a 378 nm minimum and 422 nm maximum peaks in the blue region, and 705 nm minimum and 758 nm maximum peaks in the far-red region (Fig. 3a). We found it was not possible to completely switch BV-AM1_5894ΔHK to its Pfr state, and the ratio between Pr and Pfr states remained stable after 15 min (Fig. 4a). The Pfr-state protein rapidly reverted back to its Pr state when exposed to far-red light (777 nm) (Fig. 4b) or incubated in the dark.

After dark assembly with the chromophore PCB, the AM1_5894ΔHK spectrum shares a similar, but blue-shifted peak structure to that of BV-AM1_5894ΔHK (Fig. 3). PCB-AM1_5894ΔHK has major peaks at 370 nm and 682 nm and a shoulder at approximately 625 nm (Fig. 3b). Illumination with red light initiates a partial switch to the Pfr state of PCB-AM1_5894ΔHK, with a maximum at 734 nm. The difference spectrum of Pfr minus Pr shows similar profiles and 368 nm minimum and 412 nm maximum peaks in the blue light region and 682 nm minimum and 734 nm maximum peaks in the red/far-red light region (Fig. 3b). By normalising at an absorbance of 280 nm, the absorbance intensity at 705 nm for BV-AM1_5894ΔHK is higher than that at 682 nm for PCB-AM1_5894ΔHK (Fig. 2c), indicative of a lower binding affinity of AM1_5894ΔHK for PCB compared with BV.

**Figure 5:**
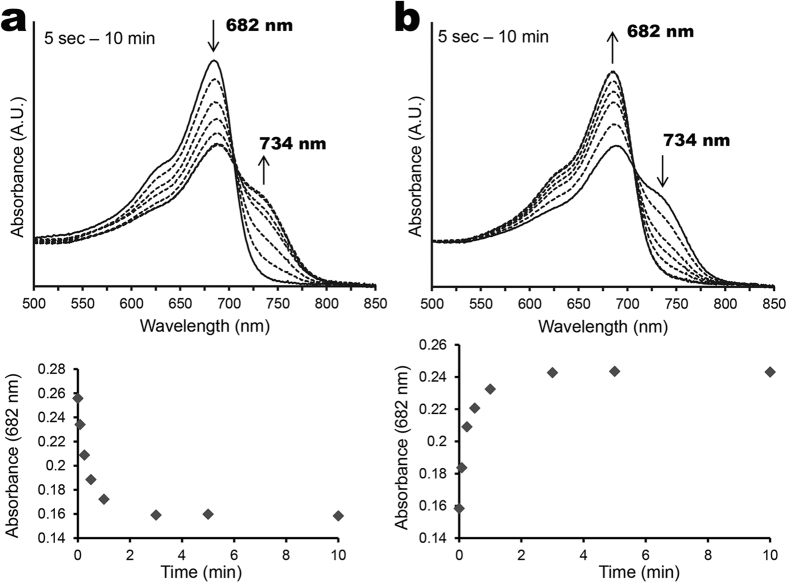
Photoconversion kinetics for PCB-AM1_5894ΔHK. (**a**) Pr state PCB-AM1_5894ΔHK was illuminated with 665 nm light for the indicated period of time. (**b**) Pfr/Pr state PCB-AM1_5894ΔHK was illuminated with 777 nm light for indicated period of time. Δ682 nm is plotted as a measure of photo-switching.

Like the BV-holoprotein, the PCB-holoprotein also could not be entirely switched to its Pfr state (Fig. 5). However, the switching kinetics between Pr-Pfr and Pfr-Pr were very different between BV-AM1_5894ΔHK and the PCB-AM1_5894ΔHK (Figs 4 and 5). The Pr-Pfr switching of BV-AM1_5894ΔHK was slow with a halflife of ~3.5 minutes while the Pfr-Pr switching was fast with a halflife of less than 5 sec (Fig. 4). Unlike the BV-AM1_5894ΔHK, the switching kinetics of PCB-AM1_5894ΔHK was approximately symmetric with one another, whether switching from Pr to Pfr or vice versa with a halflife of approximately 10 sec for the conversion (Fig. 5).

### Differences in dark relaxation and photoactive light range

**Figure 6:**
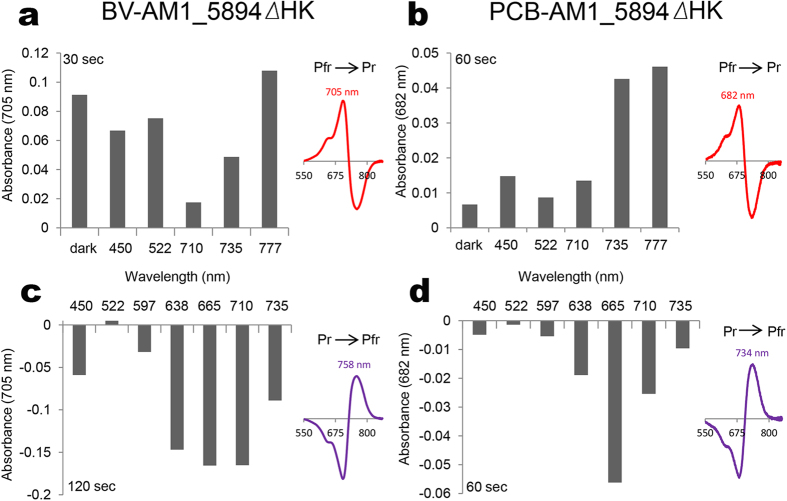
Dark and photoactive light conversion of Pfr and Pr BV-AM1_5894ΔHK (**a**,**c**) and Pfr and Pr PCB-AM1_5894ΔHK (**b**,**d**). Upper panels show Pfr to Pr photoconversion of the Pfr saturated forms of (**a**) BV- and (**b**) PCB**-AM1_5894ΔHK on exposure to darkness or saturating light emitting diodes (LEDs) at the indicated wavelength for the indicated length of time. Lower panels show Pr to Pfr photoconversion of the Pr saturated forms of (**c**) BV- and (**d**) PCB-AM1_5894ΔHK on exposure to saturating light at the indicated wavelength for the indicated time. The extent of photoconversion is shown as the change in absorbance at the Pr λ_max_, longer bars indicate higher conversion. Difference spectra insets show an example of each photoconversion.

Dark relaxation and the effects of different light wavelengths on the switching of the Pfr form of AM1_5894 to its Pr state were examined in detail for BV-AM1_5894ΔHK and PCB-AM1_5894ΔHK using different wavelength light emitting diodes (LEDs) (Fig. 6a,b, Figure S3). Maximal Pfr to Pr switching for both BV- and PCB-bound AM1_5894ΔHK was observed when exposed to far-red light (777 nm), with a similar rate of switching observed for PCB-AM1_5894ΔHK exposed to 735 nm light. Dark relaxation was rapid (approximately 85% complete after 30 s) for BV-AM1_5894ΔHK, with rates similar to those observed when it was exposed to 777 nm light, while exposure to other light wavelengths inhibited this switching (Fig. 6a). In contrast, there was only minimal dark relaxation observed for PCB-AM1_5894ΔHK after 60 s. The dark reversion of BV-AM1_5894ΔHK is faster than PCB-AM1_5894ΔHK. The low ratio of bound PCB in reconstituted PCB-AM1_5894ΔHK complexes (Fig. 2c) and the slower dark reversion (Fig. 6) support the fact that, as predicted, the BV-binding form is of the common chromophore-binding structure seen for BphPs, although it may also facilitate PCB-binding (Fig. 1).

The switching of PCB-AM1_5894ΔHK from its Pr to its Pfr state was more sensitive to light wavelength than BV-AM1_5894ΔHK. For both, 665 nm light was most efficient at inducing Pr to switch to Pfr (Fig. 6c,d). However, 710 nm and 638 nm incident light were similarly effective for BV-AM1_5894ΔHK (Fig. 6c). Unexpectedly, 450 nm light partially switches BV-AM1_5894ΔHK from Pr to Pfr states, as illustrated by both this switching and the slight reduction in the Pfr to Pr switching when comparing 450 nm-exposed BV-AM1_5894ΔHK to that left in the dark (Fig. 6a,c).

**Figure 7:**
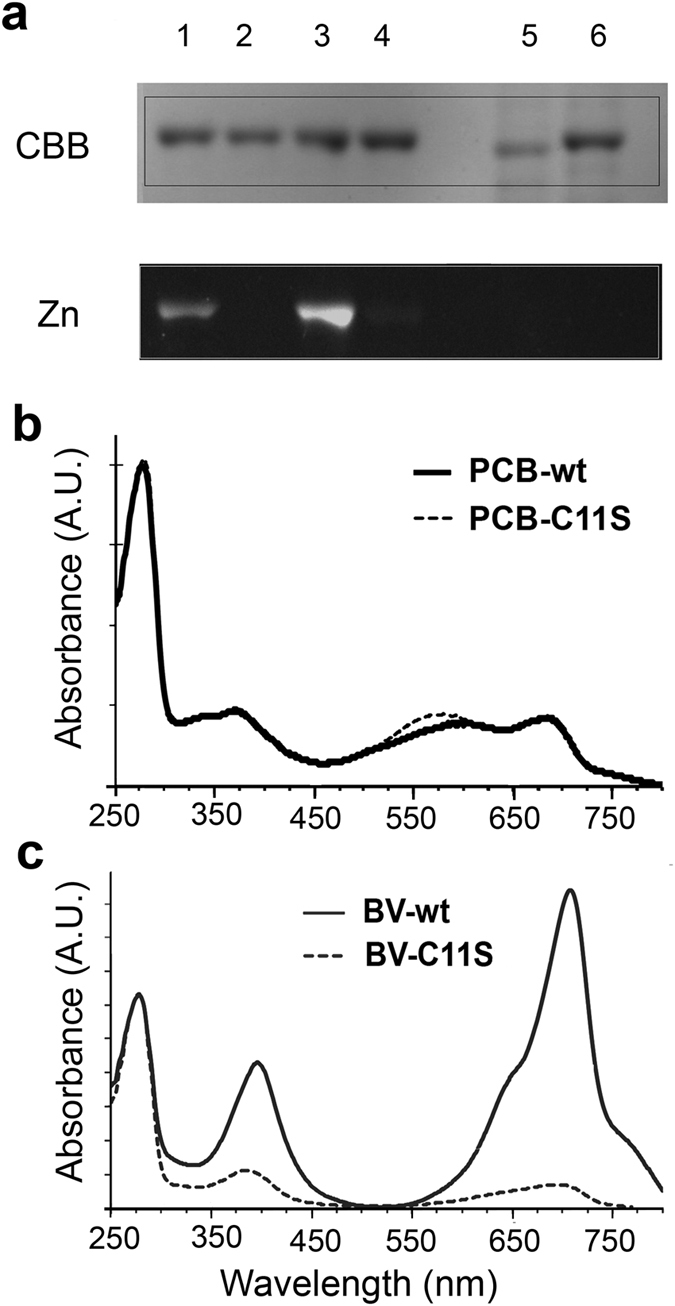
Characterization of chromophore binding to the C11S AM1_5894 mutant. (**a**) SDS polyacrylamide gel analysis on reconstituted protein complexes. 1, wt:BV-AM1_5894ΔHK; 2, C11S:BV-C11S-AM1_5894ΔHK; 3, wt:PCB-AM1_5894ΔHK; 4, C11S:PCB-C11S-AM1_5894ΔHK; 5 and 6, the purified AM1_5894ΔHKC11S protein before and after incubation in the absence of BV or PCB, respectively. Covalently bound chromophore was detected by Zn induced fluorescence (Zn). CBB, Coomassie brilliant blue. (**b**) The online spectral comparison of PCB-AM1_5894ΔHK with PCB-C11S-AM1_5894ΔHK after BioSec2000 purification; (**c**) the online spectral comparison of BV-AM1_5894ΔHK with BV-C11S-AM1_5894ΔHK after BioSec2000 purification.

It is known that a conserved Cys residue in the PAS domain of BphPs (Cys11 for AM1_5894ΔHK, Fig. 1) is the position for covalent binding of BV^5^. To determine whether Cys11 was involved in binding PCB, as well as BV, we constructed a C11S-AM1_5894ΔHK mutant, then BV or PCB was added to lysed *E. coli* containing overexpressed C11S-AM1_5894∆HK before His-tag purification. Under non-denaturing conditions, both BV and PCB seemed to elute with protein together during size exclusion chromatographic purification. However, the BV-associated complex appears much weaker than for WT AM1_5894ΔHK with a very low chromophore ratio (Fig. 7c). Although there were protein/chromophore complexes with a similar retention time to that of the WT for PCB, the spectrum of PCB-C11S-AM1_5894ΔHK was different, with an extra peak at around 555 nm, likely to be “unspecified” protein-chromophore complexes (Fig. 7b). However, no Zn-induced fluorescence was observed on the SDS-polyacrylamide gel for the C11S-AM1_5894ΔHK mutant incubated with either BV or PCB (Fig. 7a,b). Thus, our data demonstrate that Cys11 of AM1_5894 is essential for covalent binding of both BV and PCB. Both chromophores showed non-covalently associated with the C11S-AM1_5894ΔHK mutant (Fig. 7b,c), but this association was unstable. We were unable to detect any photoconversion in this mutant, associated with either BV or PCB (Figure S4).

## Discussion

*Acaryochloris* uses red-shifted Chl *d* to capture light for photosynthesis in red/far-red light enriched environments^22^. To optimize the efficiency of light capture, *Acaryochloris* dynamically regulates chlorophyll- and phycobiliprotein-based light-harvesting complexes in a manner dependent upon light intensity and quality^30^,^36^. Here, we characterized the spectral properties of AM1_5894, a newly described BphP in *Acaryochloris*. The domain architecture of AM1_5894 resembles that of other canonical BphPs, having an N-terminal photosensory core module of PAS-GAF-PHY and a C-terminal signal transduction domain (Fig. 1). However, GAF/PHY domains are phylogenetically similar to Bathy BphPs from soil dwelling bacteria (Figure S1). Almost all these Bathy BphPs have a Pfr ground state, suggesting that these soil dwelling diazotrophs use these BphPs for a non-standard response to the perception of red light^10^. Although AM1_5894 appears related to Bathy Bphs, it has a photocycle that resembles the majority of characterized phytochromes, having a Pr ground state that switches to the Pfr light-activated state when exposed to red light. AM1_5894 and Bathy BphPs also share the same signal output domain structure, with an HWE-type histidine kinase and a C-terminal receiver domain (Fig. 1). These characteristics place AM1_5894 in a cluster of BphPs termed type 2 (Figure S1)^10^ and as only type 2 BphPs share an HWE-type histidine kinase domain they are thought to share a common evolutionary origin, which is consistent with the hypothesis presented in Buchberger & Lamparter^41^. Questions clearly remain as to the origin of AM1_5894 in the *Acaryochloris* genome, as well as the similar BphP identified in *Leptolyngbya* PCC 7375.

AM1_5894 autocatalytically reconstitutes *in vitro* from the apoprotein and the chromophores BV and PCB. These holoproteins exist in two photoconvertible forms: a Pr form (dark-adapted; λ_PCB max_[Pr] 682 nm, λ_BV max_[Pr] 705 nm) and a Pfr form (light-activated state; λ_PCB max_[Pfr] 734 nm, λ_BV max_[Pfr] 758 nm). It is noted that λ_PCB max_[Pr] 682 nm of PCB-AM1_5894 is quite different from λ_PCB max_[Pr] 655 nm of Cph1, which might be a result of the different binding site or the different binding properties^12^. The ability to use different chromophores of AM1_5894ΔHK in combination with recently identified CBCRs in *Acaryochloris* provides the molecular basis for *Acaryochloris* to thrive in different ecological niches, especially in diverse light environments.

Based on sequence alignments of the GAF and PHY domains of AM1_5894 with other bacterial and plant phytochromes, we initially hypothesized that AM1_5894 would covalently bind BV, but not necessarily PCB (Fig. 1). To our surprise, Zn-induced fluorescence on SDS-polyacrylamide gels suggests that AM1_5894 forms a covalent attachment to PCB as well as BV. Our experiments with a C11S-AM1_5894ΔHK mutant suggested that PCB is covalently bound to Cys 11 of AM1_5894, although the binding mechanism is unclear (Fig. 2). The GAF domains from two CBCRs from *Acaryochloris* have also been suggested to covalently bind PCB and BV based on Zn-induced fluorescence on SDS-polyacrylamide gels^37^,^38^,^39^. Other BphPs from non-photosynthetic bacteria, such as *Deinococcus radiodurans*, *Pseudomonas syringae* and *P. aeruginosa*, also bind PCB (and PΦB) covalently, as well as their natural chromophore BV^37^,^38^. But since they do not produce PCB or PΦB, the covalent binding of PCB to these Bphs is not physiologically relevant.

When bound to BV, their spectra and Pr/Pfr switching spectral properties resemble those of BphPs, under more red-shifted sensory light wavelengths. The reconstituted BV- AM1_5894ΔHK protein complexes have Pr/Pfr absorption maxima of 705 and 758 nm, respectively. These are ~20 nm-red-shifted compared to the PCB-AM1_5894ΔHK Pr/Pfr absorption maxima of 682 and 734 nm. The cBphP from *Fremyella diplosiphone* has similar Pr/Pfr spectral profiles, with a protein domain structure resembling that of AM1_5894, *i.e*., a Cys residue in its PAS domain and no canonical Cys residue in the GAF domain that covalently binds BV but not PCB^39^. Mutational analysis suggests that the BV-binding Cys residue in the PAS domain (C24) was not the site of attachment^46^. In fact, mutation of the His residue (H267) located adjacent to the canonical PCB/PΦB binding site markedly affected BV-binding and spectral properties^5^,^47^. These data contrast with mutational analysis of BphPs in *Pseudomonas aeruginosa*, where mutation of the Cys residue in the PAS domain prevents covalent attachment of BV, while mutation of the corresponding conserved His residue does not^46^. The non-covalent attachment of PCB maintains a more conjugated chromophore, which could explain the red-shifted spectral properties in PCB-binding in cBphPs from *Fremyella diplosiphone*, compared with other PCB-phytochrome holoproteins. However, the abolition of the covalent attachment of PCB to the C11SAM1_5894ΔHK mutant indicates that this cysteine residue is the site of the PCB covalent binding (Fig. 7).

*Acaryochloris* is able to dynamically regulate its light-harvesting mechanism in response to different light conditions, reducing the level of phycobiliproteins (which absorb light at around 640 nm) in response to high levels of white light or under far-red (720 nm) light^30^,^36^. Under such conditions, phycobiliproteins are either unnecessary for light capture or unable to capture far-red light, respectively. We have shown that BV-AM1_5894 can switch from its Pr ground state to its Pfr light-activated state using light sources between 625–708 nm. It efficiently switches back to its ground state using light sources between 730–780 nm. The light wavelength range between 710–730 nm is physiologically relevant to *Acaryochloris*, since Chl *d*-protein complexes absorb maximally within this range *in vivo*. The presence of PCB-AM1_5894 in addition to CBCRs found in *Acaryochloris* provide it with an effective method of measuring changes in the light spectrum relevant to its photosynthetic absorption range, allowing it to regulate target gene expression. The ability of AM1_5894 to covalently bind both BV and PCB *in vitro* and possibly *in vivo* (both being present in *Acaryochloris*) offers much broader light detection compared with other phytochromes, giving an adaptive advantage to *Acaryochloris* in its niche environments.

Extending the search for GAF domains beyond typical Cphs and BphPs in the *Acaryochloris* genome identified at least 11 genes that encode CBCR-like GAF domains. To date, GAF domains from AM1_1186, AM1_1870 and AM1_1557, have been characterized^37^,^38^,^39^. The GAF domain of AM1_1186 binds PCB and acts as a so-called dual-Cys CBCR with a red/blue light photocycle^38^. A GAF domain from AM1_1557 (GAF domain 2) and AM1_1870 (GAF domain 3) both covalently bind PCB and BV. For both AM1_1552g2 and AM1_1870g3 bound to PCB, a red-green photocycle was observed, however this shifted to a far red-orange photocycle when PCB was replaced with BV^37^,^39^. The BV-bound recombinants have red-shifted absorption peaks at 697 nm for BV-AM1_1557g2^39^ and 706 nm for BV-AM1_1870g3^37^. Although the spectral range in which CBCRs function is considerably greater than for other phytochromes, including BphPs, it is likely that the red/far-red photocycle of AM1_5894 best overlaps with the photosynthetically-relevant spectral range for *Acaryochloris*, making it a better candidate for regulating the photoacclimation exhibited by *Acaryochloris*.

## Experimental Procedures

### Expression and purification of recombinant *Amr*BphP

One canonical phytochrome, *AM1_5894*, was identified from the *Acaryochloris marina* MBIC11017 genome. The 2544 bp *AM1_5894* open reading frame was amplified from genomic DNA of *Acaryochloris* using the following primers to allow In-Fusion^®^ (Clontech, CA, USA) cloning (forward: 5′-CGCGCGGCAGCCATATGGAAATTAGAGAACTAGCGATTT-3′, reverse: 5′-GTTAGCAGCCGGATCCTTAGGCTAAGAGTTGATGAATG-3′). The underlined sequences are 5′ overhangs specific to the *E. coli* expression vector pET15b, which introduces a hexa-histidine tag to the expressed protein. A clone harbouring a full length *AM1_5894* gene with a single amino acid substitution of Gly639 →Asp is designated as full-length AM1_5894D639G was obtained. To improve the protein overexpression yield, the 1524 bp N-terminal photosensory domain (*AM1_5894*ΔHK, lacking its histidine kinase and receiver domains) was amplified from *Acaryochloris* genomic DNA, using Phusion polymerase (New England Biolabs Ltd., UK) and the primer pair AmBph_ITA_FW (GCAGCGGCCTGGTGCCGCGCGGCAGCCATATGGAAATTAGAGAACTAGCGATTTCT) and AmBph_ITA_RV (CCAACTCAGCTTCCTTTCGGGCTTTGTTAGCAGCCGTTATTCCTGCTGCTGGTTTTCTCC). The underlined 3′ sequences correspond to pET15b sequences, which allowed assembly of the PCR product into pET15b, using a Gibson Assembly^®^ master mix (New England Biolabs). A Cys11Ser mutant of *AM1_5894*ΔHK (C11S-AM1_5894ΔHK) was synthesized as a gBlock (Integrated DNA Technologies, Corlaville, IA, USA), with the overlapping pET15b sequence. It was also assembled into pET15b using a Gibson Assembly^®^ master mix (New England Biolabs). The resulting three constructs were used to transform DH5α *E. coli* cells. The fidelity of all clones (including the point mutation that caused the Gly639Asp mutation in the full length clone) was confirmed before being used to transform Rosetta *E. coli* cells.

Rosetta *E. coli* (BL21, DE3) cultures harbouring the pET15b constructs were grown at 37 °C for 1 h and then at 21 °C until OD_600_ = 0.5 in LB media, supplemented with ampicillin and chloramphenicol at 100 μg/ml and 35 μg/ml, respectively. Expression was induced by the addition of 330 μM Isopropyl β-D-1-thiogalactopyranoside (IPTG) and cultures were grown at 10 °C for 48 h to limit aggregation of the expressed protein. The cells were harvested by centrifugation and the cell pellet resuspended in binding buffer (50 mM potassium phosphate buffer, pH 8.0, 10% (v/v) glycerol,) prior to cell lysis. Cells were lysed using a pre-chilled French Pressure Cell (GlenMills, NJ, USA) at approximately 1500 psi. His-tagged *AM1_5894* protein was purified from the clarified crude protein extract using a His Gravitrap column (GE Healthcare, Sweden) and eluted with 500 mM imidazole. In some instances, BV or PCB was added to the crude protein extract prior to purification. Eluted protein was concentrated using a 3 kDa cut-off protein concentrator (Merck Millipore, MA, USA) and rinsed in 50 mM potassium phosphate buffer in order to dilute imidazole concentration to the final of <50 mM.

### Chromophore reconstitution

Overexpressed AM1_5894ΔHK or full-length AM1_5894 (30 μM concentration of crude extract) was reconstituted *in vitro* with 100 μM of either BV (Frontier Scientific, USA) or PCB. PCB was isolated from powdered *Spirulina* (Lifestream International, NZ) by a modified methanolysis method^48^. PCB concentrations were determined spectroscopically using an extinction coefficient (ε_690nm_) of 37,900 M^−1^cm^−1^ in methanol/5%HCl^49^,^50^. For confirmation of covalent binding between the chromophore and AM1_5894 or AM1_5894ΔHK, reconstituted chromophore-protein complexes were analyzed by size exclusion chromatography using a BioSec2000 column (300 × 4.6 mm, Phenomenex) on a Shimadzu HPLC (25 mM phosphate buffer with pH 8.0 as the mobile phase, with a flow rate of 0.2 ml/min). The assembled BV-AM1_5894ΔHK or PCB-AM1_5894ΔHK was separated by SDS-polyacrylamide gel and Zn-induced fluorescence was monitored to detect protein-bound chromophores, after incubating the gel in 1 mM ZnCl_2_ for 10 min. Total protein composition for both was visualized with Coomassie Brilliant Blue staining.

### Spectral characterization for photoconversion *in vitro*

All UV/visible absorption spectra were recorded with a UV2550 spectrophotometer (Shimadzu) at room temperature. For spectral analysis, approximately 30 μM His-tag purified *AM1_5894*ΔHK (as determined by its absorbance at 280 nm) was mixed with BV or PCB at a final concentration of 20 μM and the assembly was monitored by measuring the increase in absorbance at 705 nm or 682 nm, respectively. The kinetics of switching between the Pr (a red-light absorbing form) and Pfr (a far red-light absorbing form) states of BV-AM1_5894ΔHK or PCB-AM1_5894ΔHK were monitored over time by measuring spectral changes on exposure to either 665 nm (Pfr to Pr switch) or 777 nm (Pr to Pfr) monowavelength LEDs (Figure S3). All LED applied in this experiment are dual in-line LEDs (DIP LED) powered by 1.5 V DC and 10–20 mA. The light intensity of the 665 nm LED is in the range between 3 and 8 μmol photons m^−2^ s^−1^ monitored by LI-COR light meter.

The optimal photoactive wavelengths for AM1_5894 were examined by exposure of the Pfr *AM1_5894*ΔHK bound to BV or PCB to either darkness or a series of narrow bandwidth LEDs (450, 522, 710, 735 and 777 nm). To determine the optimal wavelengths for Pr to Pfr conversion, the Pr state of BV-AM1_5894ΔHK or PCB-AM1_5894ΔHK also was illuminated with a series of narrow bandwidth LEDs (450, 522, 597, 638, 665, 710, and 735 nm). Emission spectra for these LEDs are shown in Figure S3. The times of exposure for switching are based on the kinetics of switching, corresponding to just prior to ‘saturation’ of switching. To study reversion back to its initial state, purified AM1_5894ΔHK protein were exposed to either 665 nm or 777 nm light for at least 1 min, with reversion experiments conducted 5 min apart. BV-AM1_5894ΔHK reversion from its Pr to Pfr state was activated by exposures of at least 15 min.

## Additional Information

**How to cite this article**: Loughlin, P. C. *et al*. Spectral properties of bacteriophytochrome *AM1_5894* in the chlorophyll *d*-containing cyanobacterium *Acaryochloris marina*. *Sci. Rep*. **6**, 27547; doi: 10.1038/srep27547 (2016).

## Supplementary Material

Supplementary Information
