# Supplementary material for: Spectral properties of bacteriophytochrome AM1_5894 in the chlorophyll d-containing cyanobacterium Acaryochloris marina
Source: Sci Rep. 2016 Jun 10;6:27547. doi: 10.1038/srep27547 (PMC4901347; doi:10.1038/srep27547)
Supplement: Supplementary Information [file srep27547-s1.pdf]

Spectral properties of bacteriophytochrome *AM1_5894* in the chlorophyll *d*-containing cyanobacterium *Acaryochloris marina*

Patrick C Loughlin[1], Zane Duxbury[1†], Tendo T Mukasa Mugerwa[1], Penelope MC Smith[1], Robert D Willows[2], Min Chen[1*]

[1]*School of Biological Sciences, University of Sydney, NSW 2006, Australia*

[2]*Department of Chemistry and Biomolecular Sciences, Macquarie University, NSW 2109, Australia*

[†]Current address: *The Sainsbury Laboratory, Norwich Research Park, Norwich NR4 7UH, United Kingdom*

[*]Corresponding author: School of Biological Sciences (A08), University of Sydney, NSW 2006, Australia. E-mail: min.chen@sydney.edu.au; Phone: +61 2 9036 5006; Fax: +61 2 9351 4119

Keywords: *Acaryochloris marina*, bacteriophytochrome, photosynthesis, photoregulation

Running title: Bacteriophytochrome in *Acaryochloris marina*

Supplementary

Supplementary Table

Table S1. Phytochrome sequence information used for phylogenetic analysis. Nomenclature of phytochromes is based on previous work however where this was not possible phytochromes were numbered arbitrarily. Cph and cBphP designations were based on the presence of a conserved Cys residue in the GAF (for Cphs) or PAS domain (for cBphPs) required for covalent attachment of the chromophore. The **bold sequences** are used in Figure 1 protein domain alignments.

Phytochrome family	Species and designated name	Genbank Identifier (GI)
**BphP**	***Acaryochloris* CCMEE 5140 Bph**	**498162097**
**BphP**	***Acaryochloris* MBIC 11017 AM1_5894**	**158309216**
BphP	*Agrobacterium tumefaciens* Bph1	159185005
BphP	*Agrobacterium tumefaciens* Bph2	15889444
BphP	*Agrobacterium vitis* Avp1	22173857
BphP	*Agrobacterium vitis* Avp2	221736544
BphP	*Azospirillum brasiliense* Bph1	612165886
BphP	*Azospirillum brasiliense* Bph2	503965711
BphP	*Bradyrhizobium japonicum* Bph	636815325
BphP	*Bradyrhizobium* ORS 278 Bph1	18378781
BphP	*Bradyrhizobium* ORS 278 Bph2	146191860
BphP	*Bradyrhizobium* ORS 278 Bph3	146191159
BphP	*Bradyrhizobium* STM 3843	496255428
BphP	*Deinococcus radiodurans* Bph	15807720
BphP	*Herbaspirillum seropedicae* Bph	502999494
**BphP**	***Leptolyngbya* PCC 7375 Bph**	**493561217**
BphP	*Maricaulis* JL2009 Bph	550948951
BphP	*Methylovorus* MP688 Bph	312440705
BphP	*Parvularcula oceani* Bph	671572338
BphP	*Ponticaulis koreensis* Bph	653235716
BphP	*Pseudomonas aeruginosa* Bph	553793391
BphP	*Rhizobium* MGL06 Bph	657918633
BphP	*Rhodobacter sphaeroides* Bph	17980444
BphP	*Rhodobacter sphaeroides* 2.4.1 Bph	77404754
BphP	*Rhodopseudomonas palustris* HaA2 Bph	86572318
CBCR	*Acaryochloris* MBIC 11017 CBCR	158305273

CBCR	*Nostoc* PCC 73102 CBCR	186467324
CBCR	*Synechococcus* JA-3-3Ab CBCR	86555923
**cBphP**	***Acaryochloris* CCMEE 5140 cBph**	**498164971**
cBphP	*Anabaena* ATCC 29413 cBph	499637119
cBphP	*Calothrix* PCC 7103 cBph	518327172
**cBphP**	***Fremyella* PCC 7601 cBph**	**18642523**
cBphP	*Microcoleus vaginatus* cBph	493684270
cBphP	*Nodularia* CCY 9414 cBph	493208796
**cBphP**	***Nostoc* PCC 7120 cBph**	**17230391**
Cph	*Anabaena* ATCC 29413 Cph	499639813
Cph	*Cyanothece* CCY 0110 Cph	495552723
Cph	*Fremyella* PCC 7601 Cph	18642520
Cph	Gloeocapsa PCC 7428 Cph	505002352
**Cph**	***Leptolyngbya* PCC 7375 Cph1**	**493560981**
**Cph**	***Leptolyngbya* PCC 7375 Cph2**	**493562185**
Cph	*Nodularia* CCY 9414 Cph	493212319
**Cph**	***Nostoc* PCC 7120 Cph**	**17230649**
Cph	*Nostoc* PCC 73102 Cph	186464107
**Cph**	***Synechocystis* PCC 6803 Cph1**	**16331509**
Fph	*Aspergillus nidulans* Fph	40745184
Fph	*Bipolaris maydis* Fph	39656355
Fph	*Neurospora crassa* Fph	71982796
**PHY**	***Arabidopsis* PHYA**	**16421**
**PHY**	***Arabidopsis* PHYB**	**16423**
PHY	*Arabidopsis* PHYC	37623877
PHY	*Arabidopsis* PHYD	452814
PHY	*Arabidopsis* PHYE	7268606

Supplementary Figures

Figure S1

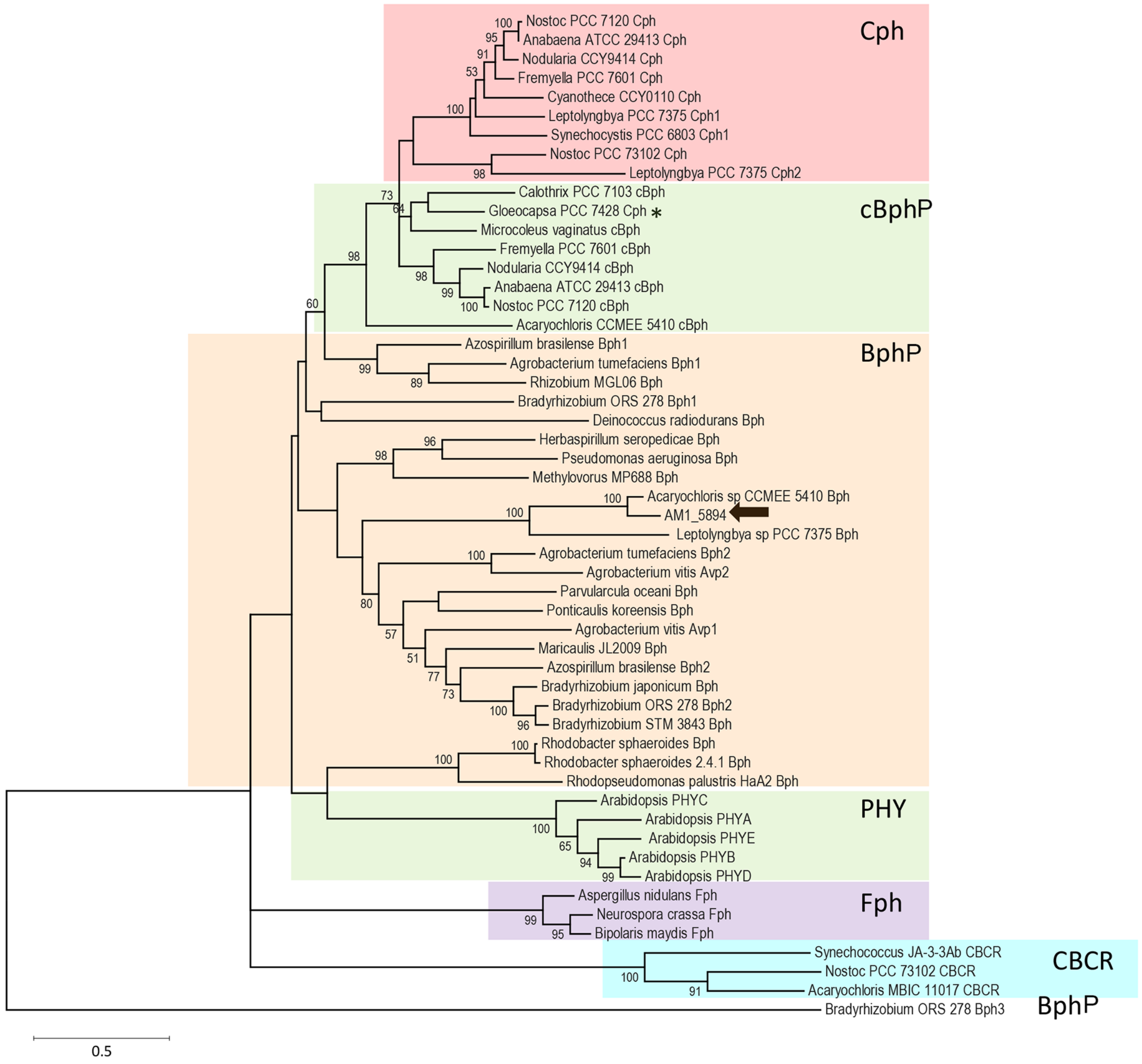

Figure S1. Maximum Likelihood phylogenetic analysis of the GAF-PHY domains of selected phytochromes. Amino acid sequences of phytochromes compiled in earlier studies were retrieved from the National Center for Biotechnology Information (NCBI) database and listed in Supplementary Table 1. Additional phytochrome candidates, including homologs in the *Acaryochloris* genome[s1], were retrieved from a BLAST search of the Genbank non-redundant (nr) protein sequences database using the PAS, GAF and phytochrome (PHY) domains of characterized phytochromes as query sequences. Conserved homologous domains were identified using SMART (smart.embl-heidelberg.de) and PFAM databases[s2,s3]. The combined GAF and PHY domains from

representative phytochrome members were aligned using ClustalX2 and compared to published alignments of phytochrome domains for verification[s4,s5]. Phylogenetic trees were constructed in the MEGA 6.06 software package[s6] using Maximum Likelihood with an LG model with rate variation among amino acids following an invariable sites gamma-distributed rates model and using the default priors (1000 generations). AM1_5894 is highlighted with an arrow. The phytochromes clustered into clades, with AM1_5894 (arrow) located in a clade with other bacteriophytochromes (BphPs). Cyanobacterial phytochrome (Cph) and cyanobacterial BV-binding bacteriophytochrome (cBphP) designations were based on the presence of a conserved Cys residue in the GAF (for Cphs) or PAS domain (for cBphPs) required for covalent attachment of the chromophore. The *Gloeocapsa* PCC 7428 Cph clustered with cBphPs. The GAF domain is named after cGMP-specific phosphodiesterases, adenylyl cyclases and FhlA proteins; while the PAS domain is named after three proteins: period circadian protein, aryl hydrocarbon receptor nuclear translocator protein and single-minded protein.

Figure S2

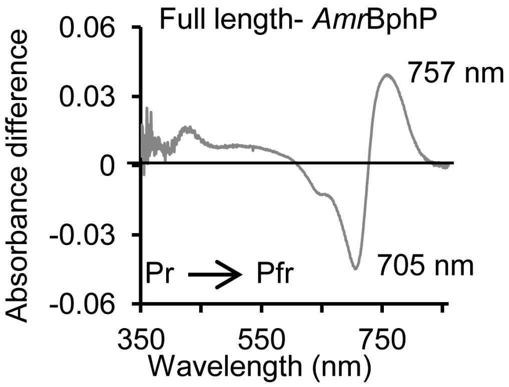

Figure S2. Pr→Pfr difference spectra of BV-assembled full length AM1_5894D639G. The reconstituted full-length BV-AM1_5894D639G shared a similar photo-conversion profile to that of the truncated protein (Figure 2).

Figure S3

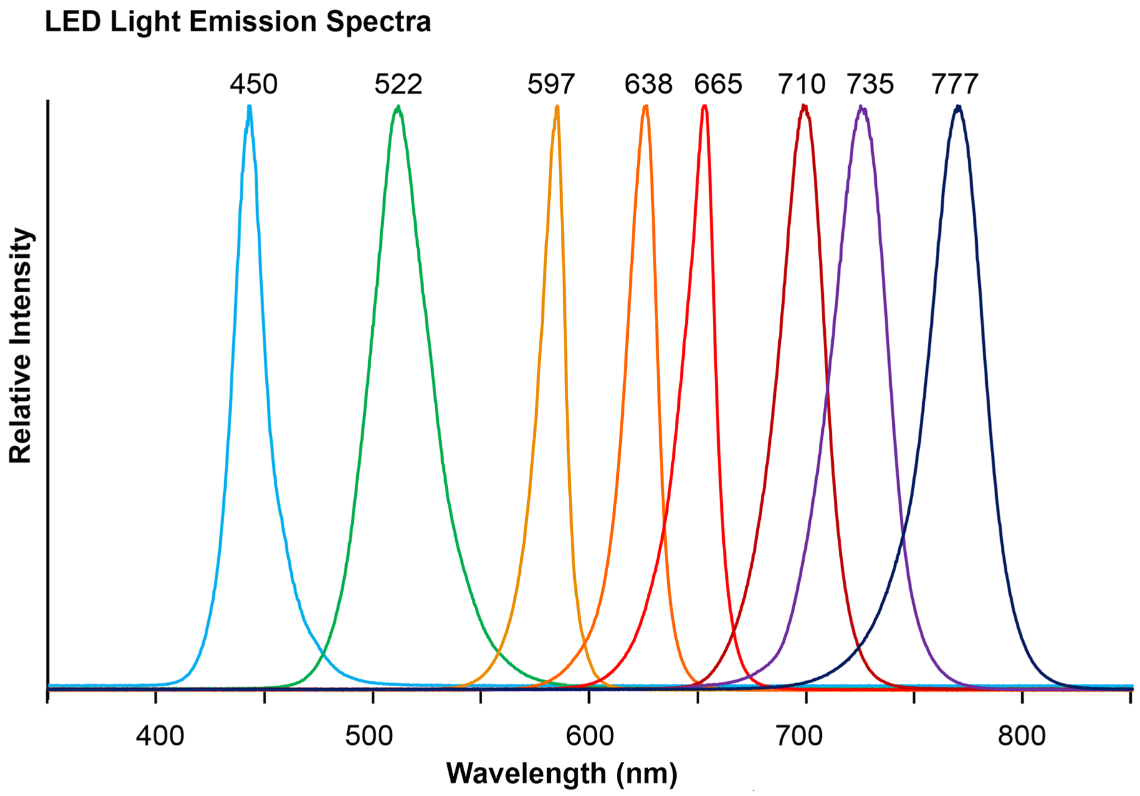

Figure S3. Emission spectra of light emitting diodes used in this study. Spectra were measured using a USB radiometer (Ocean Optics, Australia). The DIP LEDs were collected from various manufactures (Germany, Japan and P.R. China), having the LED head size of 5 mm. The photoconverting experiments were performed using a home-made DIP LEDs set-up powered by 1.5 V at 10-20 mA with the same output at ~60 mWatt for each monowavelength LEDs. The light intensity of the 665 nm LED is in the range between 3 and 8 µmol photons m^{-2} s^{-1} monitored by LI-COR light meter.

Figure S4

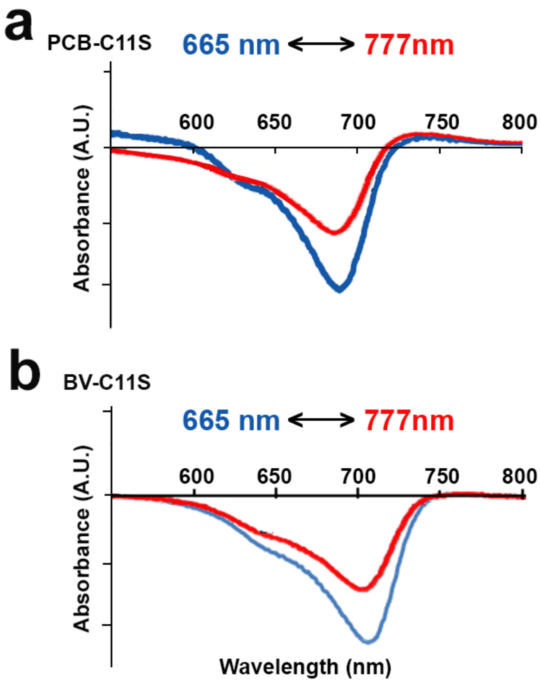

Figure S4 Spectral characterization of biliverdin (BV) and phytochyanobilin (PCB) assembled recombinant with AM1_5894C11S. (a) Difference spectra of PCB-AM1_5894 Δ HKC11S (PCB-C11S) exposed to 665 nm or 777 nm saturating light emitting diodes (LEDs), the optimal wavelengths to observe photoconversion. Blue represents the difference spectrum after 665 nm exposure spectrum was subtracted from the dark adapted recombinant mixture. The red line represents the difference spectrum after exposure to 777 nm spectrum is subtracted from 665 nm exposed sample. (b) Difference spectra of BV-AM1_5894 Δ HKC11S (BV-C11S) exposed to 665 nm or 777 nm saturating LEDs, the optimal wavelengths to observe photoconversion. Blue represents the difference spectrum after 665 nm exposure spectrum is subtracted from the dark adapted chromophore-protein mixture. The red line represents the difference spectrum after exposure to 777 nm spectrum is subtracted from 665 nm exposed sample. In all cases no photoconversion was observed and the spectral differences are likely due to the instability of the chromophore-AM1_5894 Δ HKC11S complexes.

Supplementary References

S1 Swingley, W. D. *et al.* Niche adaptation and genome expansion in the chlorophyll d-producing cyanobacterium Acaryochloris marina. *Proc. Natl. Acad. Sci. U. S. A.* **105**, 2005-2010, doi:10.1073/pnas.0709772105 (2008).

S2 Schultz, J., Milpetz, F., Bork, P. & Ponting, C. P. SMART, a simple modular architecture research tool: Identification of signaling domains. *Proc. Natl. Acad. Sci. U. S. A.* **95**, 5857-5864, doi:10.1073/pnas.95.11.5857 (1998).

S3 Letunic, I., Doerks, T. & Bork, P. SMART 6: recent updates and new developments. *Nucleic Acids Res.* **37**, D229-D232, doi:10.1093/nar/gkn808 (2009).

S4 Thompson, J. D., Higgins, D. G. & Gibson, T. J. CLUSTAL-W - improving the sensitivity of progressive multiple sequence alignment through sequence weighting, position-specific gap penalties and weight matrix choice *Nucleic Acids Res.* **22**, 4673-4680, doi:10.1093/nar/22.22.4673 (1994).

S5 Larkin, M. A. *et al.* Clustal W and clustal X version 2.0. *Bioinformatics* **23**, 2947-2948, doi:10.1093/bioinformatics/btm404 (2007).

S6 Tamura, K., Stecher, G., Peterson, D., Filipski, A. & Kumar, S. MEGA6: Molecular Evolutionary Genetics Analysis Version 6.0. *Mol. Biol. Evol.* **30**, 2725-2729, doi:10.1093/molbev/mst197 (2013).
